# Correlations of performance on a non‐spatial task and episodic memory tests

**DOI:** 10.1002/alz70857_105708

**Published:** 2025-12-25

**Authors:** Pakapon Suesatchapong, Nithit Singtokum, Pharewa Kaewmanee, Phutanik Setasartit, Sedthapong Chunamchai, Chaipat Chunharas, Anthipa Chokesuwattanaskul

**Affiliations:** ^1^ King Chulalongkorn Memorial Hospital, Thai Red Cross Society, Bangkok, Thailand; ^2^ Division of Neurology, Department of Medicine, Faculty of Medicine, Chulalongkorn University, Bangkok, Thailand; ^3^ Cognitive Clinical and Computational Neuroscience Research Unit, Faculty of Medicine, Chulalongkorn University, Bangkok, Thailand; ^4^ Chula Neuroscience Center, King Chulalongkorn Memorial Hospital, Bangkok, Thailand; ^5^ Cognitive, Clinical and Computational Neuroscience (CCCN) Research Unit, Chulalongkorn University, Bangkok, Thailand; ^6^ King Chulalongkorn Memorial Hospital The Thai Red Cross Society, Bangkok, Thailand; ^7^ Chulalongkorn Cognitive, Clinical and Computational Neuroscience, Chulalongkorn University, Bangkok, Thailand; ^8^ Dementia Research Centre, UCL Queen Square Institute of Neurology, University College London, London, United Kingdom

## Abstract

**Background:**

Alzheimer's disease (AD) is characterized by early neuropathological changes in the entorhinal cortex (EC), which plays a crucial role in spatial navigation and probably abstract space navigation. While EC‐dependent navigation tasks show promise for early AD detection, existing paradigms require extensive learning periods, limiting clinical utility. We aimed to develop and validate a novel digital non‐spatial navigation task that tests EC function without requiring prior learning, hypothesizing that performance would correlate with hippocampal‐entorhinal cortex dependent memory but not other cognitive domains

**Method:**

The study enrolled 38 participants, aged 40‐70 years‐old, who performed a computerized task navigating 2D abstract space using stripe and ellipse width dimensions. The task comprised 96 trials, each presenting three successive visual stimuli forming a trajectory, with participants selecting the correct fourth stimulus from three choices. Performance was assessed against standard cognitive measures, including MoCA, CDR, VPA delayed recall, WCST, Go/No‐Go test, and Spatial Span.

**Result:**

Performance decreased with increasing difficulty in distance deviation trials, suggesting navigation strategy usage. Trials with angular deviation showed possible prepositional strategy involvement. Task accuracy analyzed only with trials with distance deviation significantly correlated with episodic memory measures: MoCA delayed recall (*r* = 0.425, *p* = 0.008), MoCA memory index score (*r* = 0.431, *p* = 0.007), VPA delayed recall (*r* = 0.377, *p* = 0.020), and CDR memory (*r* = ‐0.535, *p* = 0.001). No significant correlations emerged with non‐memory cognitive domains. Correlations persisted after adjusting for age and education, except for VPA delayed recall.

**Conclusion:**

The study demonstrates that this novel non‐spatial navigation task probably engages EC‐hippocampal circuits, as evidenced by selective correlation with episodic memory measures. The task's design, requiring no prior learning period, represents a significant advantage over existing paradigms and potential clinical utility. Future research should investigate concurrent neural activities and evaluate diagnostic value participants with confirmed AD biomarkers.

